# Effect of the long-acting insulin analogues glargine and degludec on cardiomyocyte cell signalling and function

**DOI:** 10.1186/s12933-016-0410-9

**Published:** 2016-07-15

**Authors:** Thorsten Hartmann, Sabrina Overhagen, D. Margriet Ouwens, Silja Raschke, Paulus Wohlfart, Norbert Tennagels, Nina Wronkowitz, Jürgen Eckel

**Affiliations:** German Diabetes Center, Paul-Langerhans-Group for Integrative Physiology, Auf’m Hennekamp 65, 40225 Düsseldorf, Germany; Institute for Clinical Biochemistry and Pathobiochemistry, German Diabetes Center, 40225 Düsseldorf, Germany; German Center for Diabetes Research (DZD), Neuherberg, 85764 Munich, Germany; Department of Endocrinology, Ghent University Hospital, 9000 Ghent, Belgium; R&D Diabetes Division, Sanofi-Aventis Deutschland GmbH, 65929 Frankfurt, Germany

**Keywords:** Insulin glargine, Insulin degludec, Cardiac action, Insulin analogues

## Abstract

**Background:**

The effects of insulin on cardiomyocytes, such as positive inotropic action and glucose uptake are well described. However, in vitro studies comparing long-acting insulin analogues with regard to cardiomyocyte signalling and function have not been systematically conducted.

**Methods:**

Insulin receptor (IR) binding was assessed using membrane embedded and solubilised IR preparations. Insulin signalling was analysed in adult rat ventricular myocytes (ARVM) and HL-1 cardiac cells. Inotropic effects were examined in ARVM and the contribution of Akt to this effect was assessed by specific inhibition with triciribine. Furthermore, beating-rate in Cor.4U^®^ human cardiomyocytes, glucose uptake in HL-1 cells, and prevention from H_2_O_2_ induced caspase 3/7 activation in cardiac cells overexpressing the human insulin receptor (H9c2-E2) were analysed. One-way ANOVA was performed to determine significance between conditions.

**Results:**

Insulin degludec showed significant lower IR affinity in membrane embedded IR preparations. In HL-1 cardiomyocytes, stimulation with insulin degludec resulted in a lower Akt(Ser^473^) and Akt(Thr^308^) phosphorylation compared to insulin, insulin glargine and its active metabolite M1 after 5- and 10-min incubation. After 60-min treatment, phosphorylation of Akt was comparable for all insulin analogues. Stimulation of glucose uptake in HL-1 cells was increased by 40–60 %, with a similar result for all analogues. Incubation of electrically paced ARVM resulted for all insulins in a significantly increased sarcomere shortening, contractility- and relaxation–velocity. This positive inotropic effect of all insulins was Akt dependent. Additionally, in Cor.4U^®^ cardiomyocytes a 10–20 % increased beating-rate was detected for all insulins, with slower onset of action in cells treated with insulin degludec. H9c2-E2 cells challenged with H_2_O_2_ showed a fivefold increase in caspase 3/7 activation, which could be abrogated by all insulins used.

**Conclusions:**

In conclusion, we compared for the first time the signalling and functional impact of the long-acting insulin analogues insulin glargine and insulin degludec in cardiomyocyte cell models. We demonstrated similar efficacy under steady-state conditions relative to regular insulin in functional endpoint experiments. However, it remains to be shown how these results translate to the in vivo situation.

**Electronic supplementary material:**

The online version of this article (doi:10.1186/s12933-016-0410-9) contains supplementary material, which is available to authorized users.

## Background

In addition to skeletal muscle, liver and adipose tissue, the heart with 10,000–100,000 expressed insulin receptors (IRs) per cardiomyocyte [1] must be considered as an additional major organ affected by insulin (Ins). In cardiomyocytes, Ins modulates glucose transport, metabolism, protein synthesis, hypertrophy, contractility, beating-rate, and apoptosis [1–3].

Long-acting insulin analogues are designed to deliver a constant basal Ins supply throughout the day via the subcutaneous route resulting in improved fasting blood glucose and overall glycaemic control while reducing the risk of hypoglycaemia [4–6]. Insulin glargine (IGla) has one amino acid exchange in the A-chain and two additional arginine residues at the B-chain, resulting in an altered isoelectric point that leads to local precipitation after subcutaneous injection [7, 8]. From this depot, IGla is slowly dissolved followed by immediate biotransformation into the active metabolite M1 (IGlaM1) [9, 10]. For insulin degludec (IDeg) it has been proposed that its protracted action results from slow dissolution of subcutaneous multi-hexamer assemblies [11]. However the structure of the side chain attached to this molecule (n-16 fatty acid) [11] suggests that the protraction mode may be similar to that of insulin detemir, which binds to human serum albumin (HSA) via its fatty acid side chain, thereby protracting its duration of action by providing a ‘floating depot’ with the consequence of a reduced biological availability [12–14].

Besides the benefits of Ins analogue modification, modifying the Ins molecule may lead to an altered activation profile such as receptor signalling or pharmacodynamics and pharmacokinetics. Recently the effects of Ins analogues on the cardiovascular system gained considerable interest. So far, finalised clinical data is only available for IGla, whereas for IDeg the investigation is still ongoing. Results from the Outcome Reduction With Initial Glargine Intervention (ORIGIN) trial proved non-inferiority of IGla compared to standard care treatment in regard to cardiovascular effects (Trial number NCT00069784) [15]. Just recently the interim analysis of the dedicated cardiovascular outcome trial for IDeg (DEVOTE) (Trial number NCT01959529), requested in 2013 by the American Food and Drug Administration (FDA) [16] suggested non-inferiority to IGla, leading to approval of IDeg for the US market with a post marketing commitment to provide further non-inferiority data in major adverse cardiovascular events [17]. These clinical trials were designed to compare systemic metabolic effects of the analogues in patients. However, strong evidence for tissue-specific action of Ins exists [18] and to the best of our knowledge, there are no in vitro studies published which elucidate and compare the effect of different long-acting Ins analogues on cardiomyocyte cell models. Thus, data regarding the signalling and function of Ins analogues in cardiomyocyte cell models might shed light on potential differences of these drugs in relation to their cardiac action. Therefore, we compared the impact of IGla, IGlaM1, and IDeg to Ins in regard to signalling, contractility and anti-apoptotic potency in HL–1 cardiomyocytes, ARVM, H9c2-E2, and Cor.4U^®^ cells. Our data show a very similar cardiomyocyte action profile for both IGla and IDeg, at least under steady-state conditions.

## Methods

### Cell culture

The cardiac mouse cell line HL-1, a cell line derived from the AT-1 mouse atrial cardiomyocyte tumour lineage [19], was kindly provided by Dr. W.C. Claycomb (Louisiana State University, New Orleans, LA, USA). HL-1 cells were cultivated in Claycomb medium containing 10 % fetal calf serum (FCS), 100 µM norepinephrine and 4 mM l-glutamine (all from Sigma-Aldrich, Munich, Germany) on gelatine/fibronectin coated plates. H9c2 cells (ATCC CRL-1446), stably transfected with the human IR in our laboratory [20] (H9c2-E2) were cultivated in DMEM, low glucose containing 10 % FCS, 1 % non–essential amino acids and 600 µg/ml G418 (all from Invitrogen, Carlsbad, CA, USA). Commercially available iPS-derived human cardiomyocytes (Cor.4U^®^) (Axiogenesis, Cologne, Germany) were cultured in Cor.4U^®^ Complete Medium containing 10 % FCS on fibronectin–coated 96-well E-Plate (Acea Biosciences, San Diego, CA, USA). The medium was changed twice daily. All cells were incubated at 37 °C with 5 % CO_2_ in a humidified incubator.

### Competition binding experiments on membrane embedded and solubilised insulin receptor preparations

Isolation of insulin receptor embedded plasma membranes (M-IR) and competition binding experiments were performed as previously described [21]. Briefly, CHO-cells overexpressing the IR were collected and re-suspended in ice-cold 2.25 STM buffer (2.25 M sucrose, 5 mM Tris–HCl pH 7.4, 5 mM MgCl_2_, complete protease inhibitor) and disrupted using a Dounce homogenizer followed by sonication. The homogenate was overlaid with 0.8 STM buffer (0.8 M sucrose, 5 mM Tris–HCl pH 7.4, 5 mM MgCl_2_, complete protease inhibitor) and ultra-centrifuged for 90 min at 100,000*g*. Plasma membranes at the interface were collected and washed twice with phosphate buffered saline (PBS). The final pellet was re-suspended in dilution buffer (50 mM Tric-HCl pH 7.4, 5 mM MgCl_2_, complete protease inhibitor) and again homogenised with a Dounce homogenizer. Competition binding experiments were performed in a binding buffer (50 mM Tris–HCl, 150 mM NaCl, 0.1 % BSA, complete protease inhibitor, adjusted to pH 7.8) in 96-well microplates. In each well 2 µg isolated membrane were incubated with 0.25 mg wheat germ agglutinin polyvinyltoluene polyethylenimine scintillation proximity assay (SPA) beads. Constant concentrations of [^125^I]-labelled human Ins (100 pM) and various concentrations of respective unlabelled Ins (0.001–1000 nM) were added for 12 h at room temperature (23 °C). The radioactivity was measured at equilibrium in a microplate scintillation counter (Wallac Microbeta, Freiburg, Germany).

Binding on a freshly solubilised IR preparation (S-IR) was performed as previously described [22] with some modifications. Aliquots of membranes were incubated at 4 °C for 30 min in a solubilisation buffer (20 mM HEPES–NaOH, 100 mM NaCl, 10 mM MgSO4, 1 % (w/v) n-Dodecyl-ß-d-maltoside (Sigma-Aldrich, Munich, Germany), adjusted to pH 7.8 and Complete TM Protease Inhibitor cocktail). Thereafter, ultra-centrifugation was performed at 100,000*g* for 30 min and 4 °C to remove non–solubilised debris. Protein concentration in the supernatant was adjusted to 0.15 mg/ml with binding buffer (100 mM HEPES–NaOH, 100 mM NaCl, 10 mM MgSO_4_, 0.025 % (v/v) Tween-20, adjusted to pH 7.8 and complete TM protease inhibitor cocktail). To streptavidin SPA beads (5 mg in 1000 ml binding buffer), 50 µl of an anti-IR alpha-antibody 83-7 (Abcam, Cambridge, UK) was added. After incubation for 30 min, SPA beads were once washed and finally re-suspended in 500 µl binding buffer. A solution of solubilised receptor (1 ml, 0.15 mg/ml) was added and incubated for further 60 min, before washing and resuspension in 1.5 ml. Subsequently, 100 µl re-suspended IR-Antibody-SPA beads (containing 10 µg total protein) were mixed with 50 µl [^125^I]-labelled insulin tracer (100 pM) and 50 µl non-radioactive Ins (0.001 – 1000 nM), incubated for 12 h at room temperature (23 °C) under shaking, centrifuged for 2 min and measured in the scintillation counter (Wallac Microbeta, Freiburg, Germany).

### Effect of insulin and insulin analogues on contractility of primary adult rat ventricular cardiomyocytes

Adult rat ventricular cardiomyocytes (ARVM) were isolated from wild-type Lewis rats (Lew/Crl) as previously described [23]. ARVM were cultivated 3 h in Medium 199 with Hanks’ balanced salts containing 5 mM creatin, 2 mM carnitine and 5 mM taurine supplemented with 10 % FCS and 1 % insulin/transferrin/selene on laminin–coated dishes (ibidi GmbH, Martinsried, Germany). Subsequently, ARVM were cultivated over-night in DMEM/F12 containing 33 µM biotin and 17 µM pantothenate (Invitrogen, Carlsbad, CA, USA). Prior to measurement, ARVM were pre-incubated for 5 min with 100 nM of Ins (porcine Ins, Cat. No.: I5523, Sigma-Aldrich, Munich, Germany), IGla, IGlaM1 or IDeg (provided by Sanofi-Aventis, Frankfurt a.M., Germany) in modified Tyrodes solution: 125 mM NaCl; 1.2 mM KH_2_PO_4_; 2.6 mM KCl; 1.2 mM MgSO_4_*7H_2_O; 1 mM CaCl_2_*2H_2_O; 10 mM Glucose; 10 mM HEPES; adjusted to pH = 7.4 prior to measurement. Furthermore, untreated ARVM or ARVM treated with 10 nM isoproterenol (Sigma-Aldrich, Munich, Germany) were immediately measured. ARVM were paced with bipolar pulses in a contractility and fluorescence system (IonOptix, Milton, MA, USA) at 15 V, 1 Hz, 0.5 ms, at 37 °C for up to 10 min and 10–14 contractions of at least 10 rod–shaped ARVM per condition were recorded. Sarcomeric shortening, shortening rate and re-lengthening rate were calculated using the IonWizard software (IonOptix, Milton, MA, USA). To determine the role of Akt for the positive inotropic effect of Ins and the analogues, ARVM were pre-treated with 10 µM of the specific Akt–inhibitor triciribine (Sigma-Aldrich, Munich, Germany) for 30 min in contraction buffer. Afterwards, ARVM were treated as described above. After 30 min treatment with 10 µM triciribine, ARVM viability was assessed by incubating the cells with 0.1 % trypan blue in PBS for 5 min. Microscopic pictures were taken randomly with at least 10 pictures per condition. As a positive control 200 µM H_2_O_2_ was utilised. For each condition at least 400 cells were counted per experiment.

### Immunoblotting

ARVM and HL-1 cells were treated as indicated and lysed in buffer containing 50 mM HEPES (pH 7.4) (PromoCell, Heidelberg, Germany), 1 % Triton X-100 (Sigma-Aldrich, Munich, Germany), PhosSTOP and CompleteTM protease inhibitor cocktail (Roche, Basel, Switzerland). After incubation for 2 h at 4 °C, the suspension was centrifuged at 10,000*g* for 15 min. 5 μg protein sample of the total cell lysate was separated by SDS/PAGE (10 % gel) and transferred to a polyvinylidene fluoride (PVDF) membrane. Membranes were blocked in tris-buffered saline (TBS) containing 0.1 % tween 20 and 5 % (w/v) non-fat dried skimmed milk powder and incubated overnight with anti-phospho Akt(Ser^473^) antibody, anti-phospho Akt(Thr^308^), anti-GAPDH antibody (all Cell Signalling Technology, Danvers, MA, USA) or anti-tubulin antibody (Abcam, Cambridge, UK). After washing, membranes were incubated with appropriate horseradish peroxidase-coupled secondary antibody and processed for enhanced chemiluminescence (ECL) detection using Immobilion horse radish peroxidase (HRP) substrate (Millipore, Darmstadt, Germany). Signals were visualised and evaluated on a VersaDoc 4000 MP Bio-Rad Laboratories work station and analysed by Quantity One analysis software (version 4.6.7) (both Bio-Rad Laboratories, Hercules, CA, USA).

### Impedance measurement in human Cor.4U^®^ cells

Cor.4U^®^ cardiomyocytes were seeded at a density of 30,000 cells per well. From day 3 on the cells were cultured in Iscove’s Basal Medium containing 1 % GlutaMAX supplement (both Life Technologies, Carlsbad, CA, USA) and 2 µg/ml Ciprobay (Bayer, Leverkusen, Germany). Treatment with 500 nM of the designated Ins or analogue or 100 nM isoproterenol was started at day 4 after seeding. Impedance of each well was measured with the RTCA Cardio xCELLigence Analyser [24] (Acea Biosciences, San Diego, CA, USA) during the whole experiment. Beating-rate and cell index were analysed by RTCA cardio software (Acea Biosciences, San Diego, CA, USA).

### Glucose uptake in HL-1 cardiomyocytes

HL-1 cells were seeded at a density of 400,000 cells per well in a 12-well plate. Glucose uptake was measured in serum-starved HL-1 cells, either kept untreated or exposed for 60 min to 200 nM Ins and the analogues, respectively. Subsequently 0.12 mM deoxy–d–glucose (Sigma-Aldrich, Munich, Germany) with 0.055 mCi 2–deoxy–D–[^14^C]glucose (PerkinElmer, Waltham, MA, USA) was added to the cells. After 10 min incubation the uptake was terminated by repeated washing with ice cold PBS. Afterwards, the cardiomyocytes were lysed with lysis buffer containing 1 % SDS and 200 mM NaOH. Incorporated glucose was measured by scintillation counting of the cell lysates in a liquid scintillation counter (Beckman Coulter, Pasadena, CA, USA). Values were corrected for non-specific uptake as measured by incubation with L-[^14^C]glucose (PerkinElmer, Waltham, MA, USA).

### Caspase 3/7 activity assay

To assess the anti-apoptotic effect of Ins and its analogues, H9c2-E2 cells were seeded in a density of 5000 cells per well of a 96-well plate. The next morning cells were treated with 100 nM of the respective Ins either in the presence or absence of 800 µM of H_2_O_2_ for 2 h. Caspase 3/7 activity was then measured by the caspase-Glo^®^ 3/7 assay system (Promega, Madison, Wisconsin, USA) as described in the manual. After 2 h incubation period caspase 3/7 activity was analysed by measuring the luminescence in an Infinite 200 plate reader (Tecan, Männedorf, Switzerland).

### Statistical analysis

Results are expressed as mean values ± SEM of at least three independent experiments. For statistical analysis Graphpad Prism v5.00 (Graphpad Software, San Diego, CA, USA) was used. One-way ANOVA was performed to determine significance between conditions, with level of significance chosen at p < 0.05. In case of IC_50_ determinations for binding, a non-parametric Kruskal–Wallis testing was performed, again with level of significance chosen at p < 0.05.

## Results

### Insulin degludec shows lower binding affinity and slower onset of insulin signalling

**Fig. 1:**
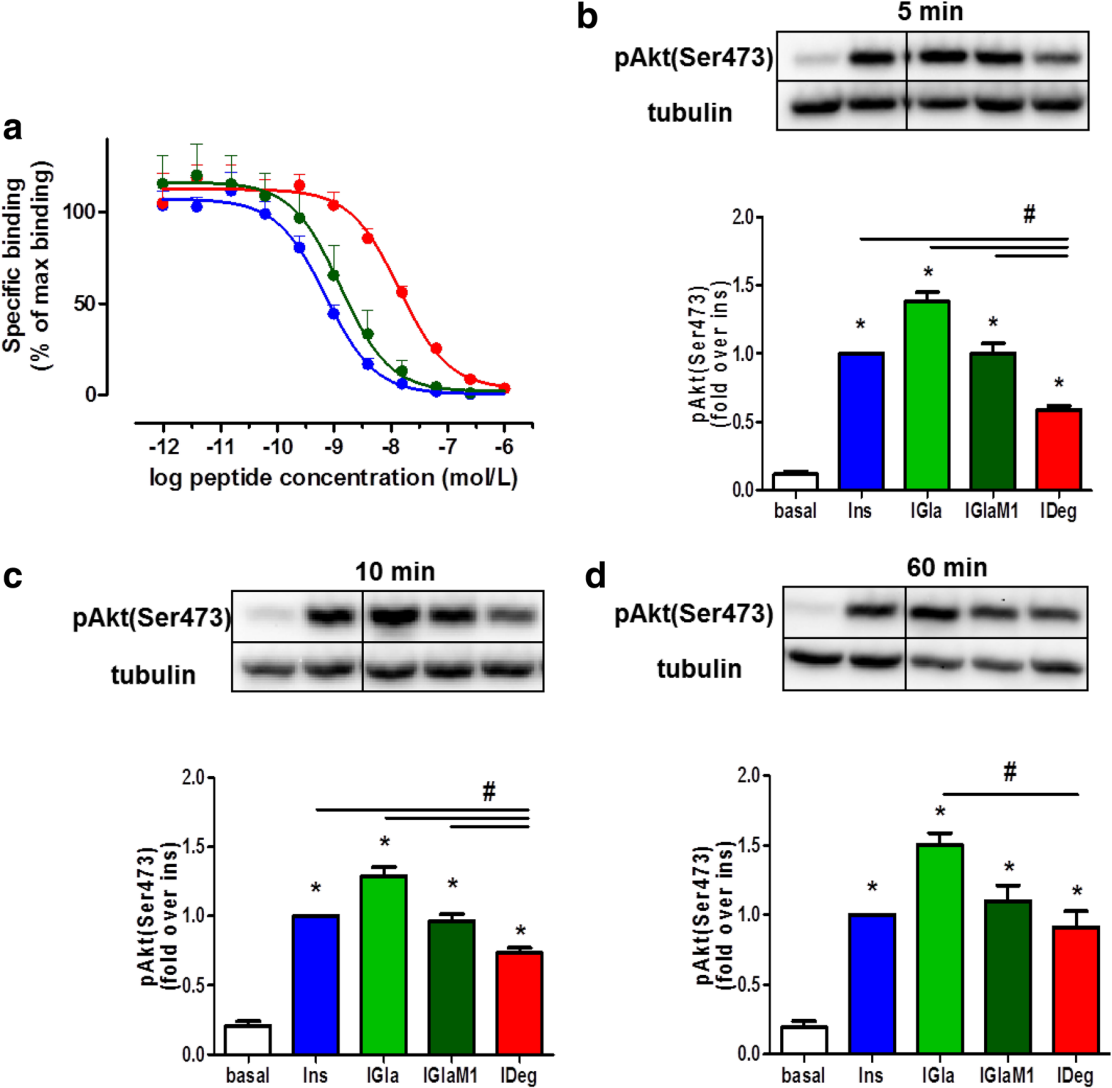
Binding affinity and Akt signalling of long-acting insulin analogues. **a** Membrane embedded insulin receptor preparations were used to analyse binding of Ins, IGlaM1 and IDeg in a competition binding assay, as described in [21]. Percentage of binding is normalised to maximum binding of [^125^I]-labelled human insulin. Data represent mean values ± SEM, n = 4-5. **b**–**d** HL-1 cells were used to assess the onset of insulin action by treatment with 200 nM for 5 (**b**); 10 (**c**) or 60 min (**d**) with insulin or insulin analogues. Phosphorylation of Akt(Ser^473^) was assessed by Western blot analysis. Data are normalised to tubulin levels. Representative blots are shown. Data represent mean values ± SEM, n = 4–5, *p < 0.05 vs. basal,^ #^p < 0.05 vs. IDeg. Regular insulin *Ins*, insulin glargine *IGla*, active metabolite of insulin glargine *IGlaM1*, insulin degludec *IDeg*

First we compared the binding affinities of Ins, IGlaM1 and IDeg with a competitive binding assay using M-IR (Fig. 1a) and S-IR (Additional file 1: Figure S1). The analysis revealed an IC_50_ value of 0.83 ± 0.07 nmol/L for Ins in M-IR binding assays (Table 1). The IC_50_ of IGlaM1 and IDeg were 1.8- and 23.6-fold higher compared to Ins, reflecting a substantial lower binding affinity for IDeg. However, in S-IR binding assays we observed higher binding affinities for all tested insulins. The IC_50_ value for Ins was 0.58 ± 0.10 nmol/L (Additional file 2: Table S1). Under these conditions binding of IDeg improved with a 5.4-fold higher IC_50_ compared to Ins. Next, to compare the activation of the Ins signalling cascade by Ins, IGla, IGlaM1 and IDeg, we assessed the Ser^473^ phosphorylation of Akt by western blot analysis in HL-1 cardiomyocytes (Fig. 1b–d). The time-course revealed a significantly lower Akt(Ser^473^) phosphorylation level after 5 and 10 min treatment with IDeg, compared to Ins and the other Ins analogues at 200 nM. After 60 min treatment IDeg showed a similar Akt(Ser^473^) phosphorylation level compared to Ins and IGlaM1; however, it was still significantly lower compared to IGla (Fig. 1d). The same kinetic was observed for Akt(Thr^308^) phosphorylation (Additional file 3: Figure S2).

**Table 1:** Summary of competition binding assay using M-IR

Insulin	IR affinity IC_50_ (nmol/L)	P value vs. Ins
Ins	0.83 ± 0.07	–
IGlaM1	2.15 ± 0.61	0.2350
IDeg	19.59 ± 1.10	<0.0001

Data represent mean ± SEM. All insulins were tested in at least four independent experiments on different days. Binding values within a single experiment were obtained in quadruplicates per insulin and averaged for each experiment

*Ins* Regular insulin; *IGlaM1* active metabolite of glargine; *IDeg* insulin degludec

### Positive inotropic effect of insulin and long-acting insulin analogues

**Fig. 2:**
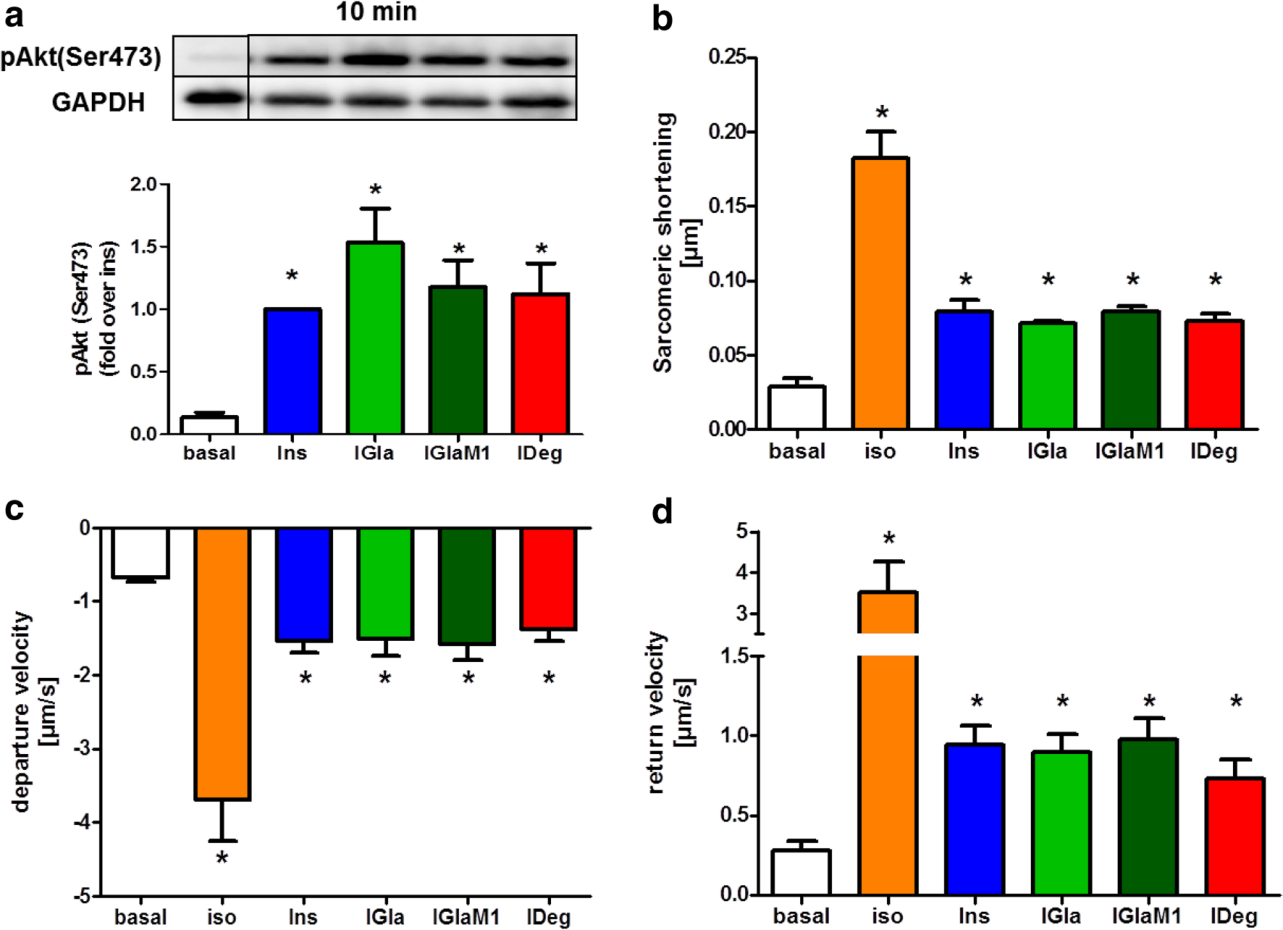
Effect of insulin and insulin analogues on Akt signalling and cardiac contraction in ARVM. Adult rat ventricular cardiomyocytes (ARVM) were treated for 10 min with 100 nM insulin or insulin analogues to investigate the insulin signalling pathway in these cells (**a**). ARVM were isolated by enzymatic digestion, starved overnight and treated with 100 nM insulin or insulin analogues or 10 nM isoproterenol as positive control. ARVM were paced at 1 Hz, 15 V, 0.5 ms with the IonOptix Myopacer Cell Stimulator System to assess the sarcomeric shortening (**b**), departure velocity (**c**) and return velocity (**d**). In each condition at least 10 contractions of 10-14 cardiomyocytes were measured under steady-state conditions. Data represent mean values ± SEM, n = 4, *p < 0.05 vs. basal. Regular insulin *Ins*, isoproterenol *iso*, insulin glargine *IGla*, active metabolite of insulin glargine *IGlaM1*, insulin degludec *IDeg*

To confirm a full activation of Akt in ARVM under experimental conditions, we first analysed the Akt (Ser^473^) and Akt (Thr^308^) activation after 10 min incubation with Ins and the different analogues. We observed a comparable Akt response under all conditions (Fig. 2a; Additional file 4: Figure S3). Next, we compared the positive inotropic effect of the different Ins analogues to Ins in freshly isolated ARVM. ARVM pre-treated with 100 nM Ins, IGla, IGlaM1 and IDeg for 5 min, respectively showed a significant increase in sarcomeric shortening (~2.5-fold), which describes the total sarcomeric shortening per sarcomere in µm (Fig. 2b). Departure- (~twofold) (Fig. 2c) and return velocity (~threefold) (Fig. 2d), which describes the shortening rate and re-lengthening rate of single sarcomeres in µm/s, are significantly increased compared to the control situation (Fig. 2). As can be seen from the data, an equipotent positive inotropic effect was observed for all analogues.

### The positive inotropic effect of insulin and long-acting insulin analogues is Akt dependent

**Fig. 3:**
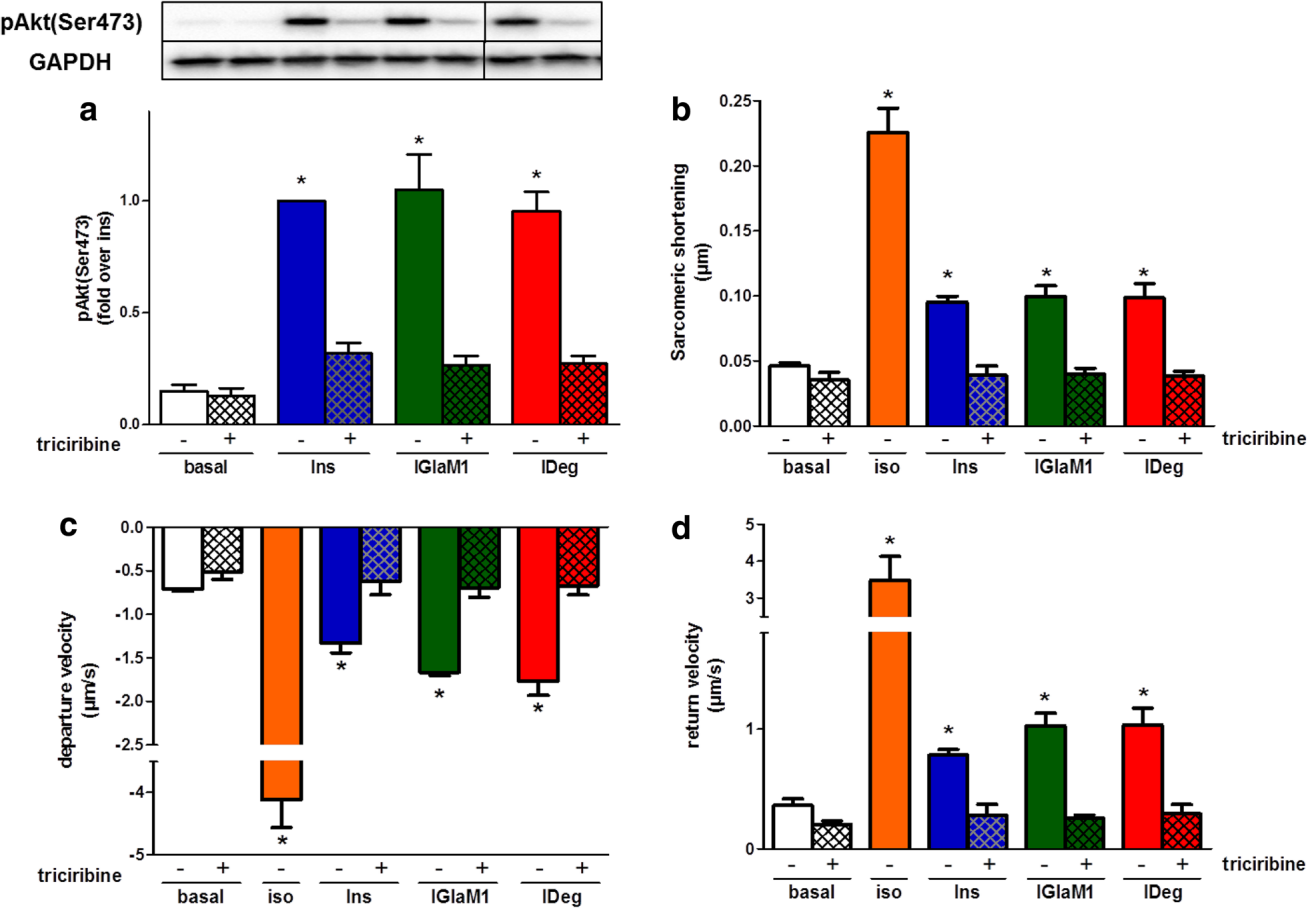
Impact of Akt inhibition on insulin-induced cardiac contraction. Adult rat ventricular cardiomyocytes (ARVM) were analysed either without pre-treatment (*blank bars*) or pre-treated with 10 µM of the specific Akt-inhibitor triciribine (*filled bars*) for 30 min. Subsequently, ARVM were treated with either 100 nM insulin or insulin analogues for 10 min to investigate the insulin signalling pathway after triciribine treatment (**a**). Furthermore, ARVM were treated with 100 nM of insulin or insulin analogues or 10 nM isoproterenol and paced at 1 Hz, 15 V, 0.5 ms with the IonOptix Myopacer Cell Stimulator System to assess the sarcomeric shortening (**b**), departure velocity (**c**) and return velocity (**d**). In each condition at least 10 contractions of 10–14 cardiomyocytes were measured. Data represent mean values ± SEM, n = 3–5, *p < 0.05 vs. basal (treated). Regular insulin *Ins*, isoproterenol *iso*, active metabolite of insulin glargine *IGlaM1*, insulin degludec *IDeg*

To assess the role of Akt in the insulin-induced inotropic effect, we treated ARVM with the specific Akt-inhibitor triciribine. Triciribine in a concentration of 10 µM did not affect cell viability as determined by trypan blue staining (Additional file 5: Figure S4). After 30 min pre-incubation with triciribine Ins signalling (Fig. 3a; Additional file 4: Figure S3) was completely abolished with all tested insulins. In line with the signalling data, the positive inotropic effect of Ins and the different insulin analogues was completely abolished at the level of all cardiac contraction parameters, such as sarcomeric shortening (Fig. 3b), departure velocity (Fig. 3c) and return velocity (Fig. 3d).

### Both insulin glargine and insulin degludec increase the beating rate of human cardiomyocytes

**Fig. 4:**
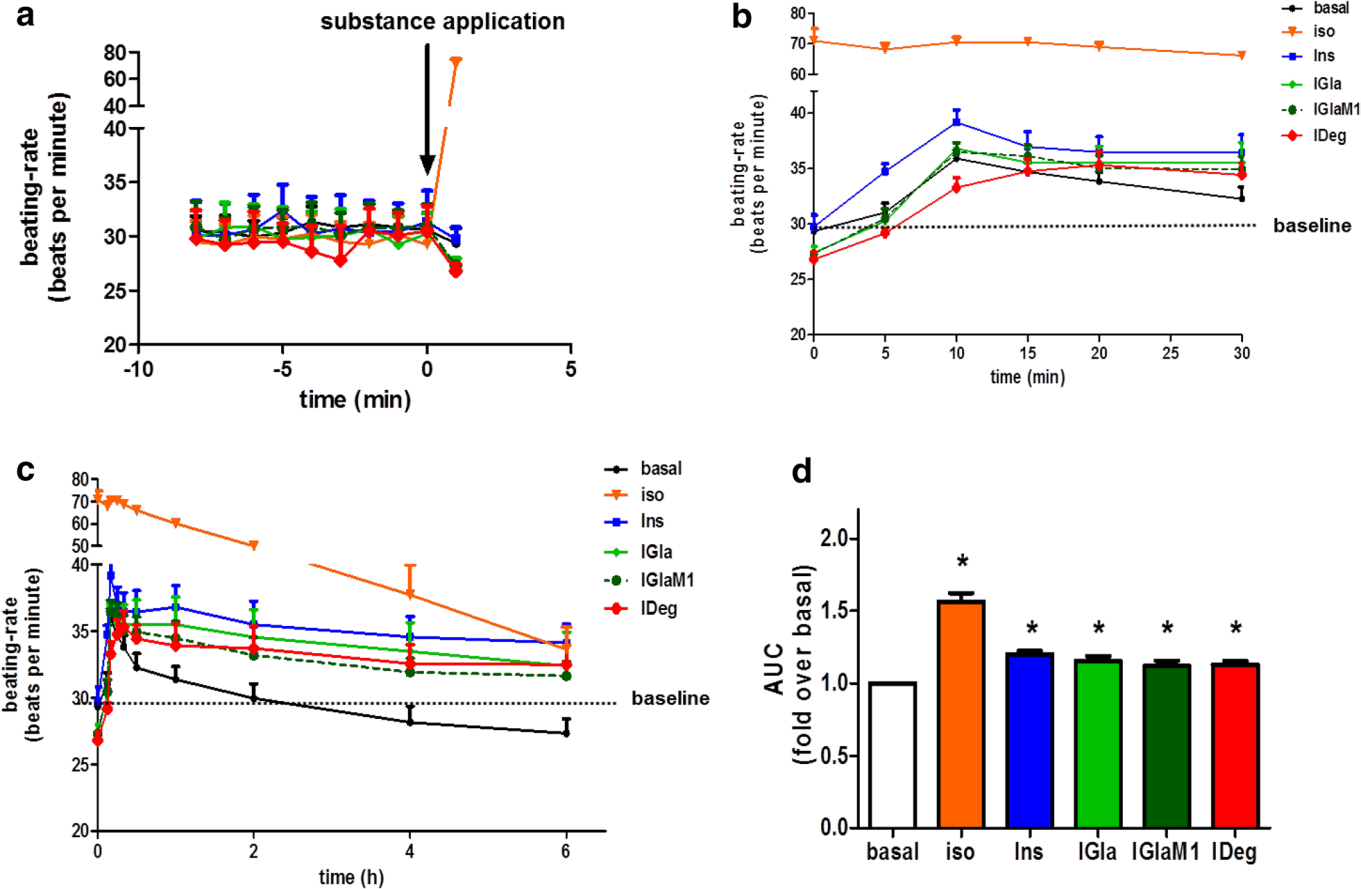
Effect of insulin glargine and insulin degludec on the beating-rate of human iPSC-derived cardiomyocytes. Commercially available human iPSC-derived cardiomyocytes (Cor.4U^®^) were used to assess the long-term effect of insulins on the beating-rate (**a**–**d**). Experiments were performed at day 4 after seeding. Cells were treated with 500 nM of insulin or insulin analogues or 100 nM isoproterenol, respectively. The beating-rate was measured for 6 h. **a** Basal beating-rate prior to substance application. **b** Beating-rate of the first 30 min after substance application. **c** 0.5–6 h time course of beating-rate in Cor.4U^®^ cells after substance application. **d** Area under the curve for the 6 h beating-rate measurement. Data represent mean values ± SEM, n = 4, *p < 0.05 vs. basal. Regular insulin *Ins*, isoproterenol *iso*, insulin glargine *IGla*, active metabolite of insulin glargine *IGlaM1*, insulin degludec *IDeg*

To investigate long-term effects of insulin and its analogues on the beating–rate of human iPSC-derived cardiomyocytes (Cor.4U^®^) we used an impedance-based measurement approach. To determine baseline values, beating–rate prior to substance application was analysed. In all measured conditions the mean beating-rate was around 30 beats per minute (bpm) (Fig. 4a). Since we did not observe any effects with 100 nM of the respective insulins, we used 500 nM for the treatment of the Cor.4U^®^ cells. After substance application Ins, IGla and IGlaM1 reach their maximum beating-rate after 10 min, whereas IDeg reaches its maximum with a delay after 20 min (Fig. 4b) and subsequently, the increased beating-rate remained stable for up to 6 h (Fig. 4c). For better comparison of the increased beating-rate in each condition we calculated the area under the curve for the whole measurement (Fig. 4d). The analysis revealed a significant increase in the beating-rate for Ins, IGla, IGlaM1 and IDeg.

### Both insulin glargine and insulin degludec increase glucose-uptake to the same extent as regular insulin

**Fig. 5:**
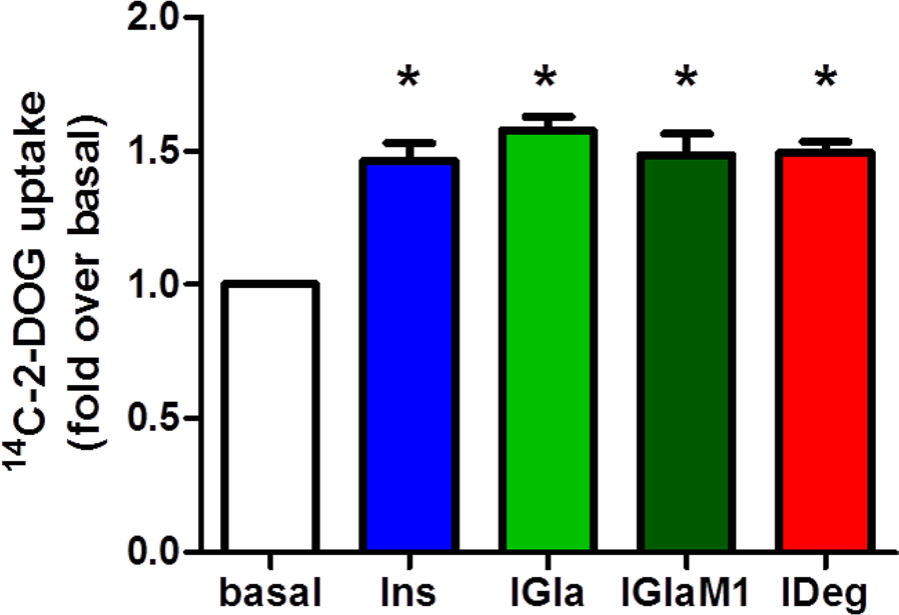
2-deoxy-d-glucose uptake in HL-1 cells after insulin stimulation with regular insulin and long-acting insulin analogues. HL-1 cells were used to assess the effect of insulin, insulin glargine, the active metabolite M1 and insulin degludec on 2-deoxy-d-glucose uptake. The cells were pre-treated with 200 nM of the indicated insulin for 1 h. Subsequently, the cells were exposed to radioactive labelled mix containing 2–deoxy–d–glucose and 2–deoxy–d–[1–^14^C]glucose for 10 min at 37 °C. Data represent mean values ± SEM, n = 4, *p < 0.05 vs. basal. Regular insulin *Ins*, insulin glargine *IGla*, active metabolite of insulin glargine *IGlaM1*, insulin degludec *IDeg*

Since glucose metabolism is important for cardiomyocyte function, we compared the potency of Ins, IGla, IGlaM1 and IDeg to stimulate glucose uptake in HL–1 cells. Glucose uptake was significantly increased after 200 nM of IGla, IGlaM1 and IDeg treatment (between 1.46- and 1.58-fold) and no difference between the insulins has been detected (Fig. 5).

### Anti-apoptotic efficiency of insulin glargine and insulin degludec is comparable to regular insulin

**Fig. 6:**
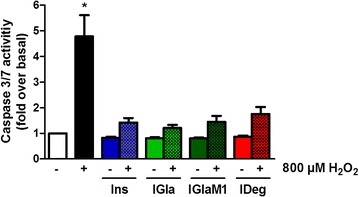
Anti-apoptotic potency of long-acting insulin analogues in the presence of H_2_O_2_. H9c2 cardiomyocytes overexpressing the human insulin receptor (H9c2-E2 cells) were treated for 2 h with 100 nM of insulin and insulin analogues in the presence or absence of 800 µM H_2_O_2_ to evaluate the cardio protective effects of insulin and its analogues. Caspase 3/7 activity was measured using the promega caspase 3/7 Glo Assay. Each condition was performed in quadruplicates. Data represent mean values ± SEM, n = 5–6, *p < 0.05 vs. basal. Regular insulin *Ins*, insulin glargine *IGla*, active metabolite of insulin glargine *IGlaM1*, insulin degludec *IDeg*

Next we evaluated the anti-apoptotic potency of the long-acting insulin analogues in the rat cardiomyocyte cell line H9c2 overexpressing the human IR (H9c2-E2). For this purpose, we treated the cells with a combination of either insulin or the different analogues in the presence of H_2_O_2_, and subsequently measured caspase 3/7 activation. After exposure to H_2_O_2_ we found a significant induction of caspase 3/7 activity in H9c2-E2 cell (4.8-fold) (Fig. 6). However, the combination of Ins and H_2_O_2_ decreased caspase 3/7 activity up to 70.3 % compared to H_2_O_2_ treatment alone, and was not significant anymore. A similar effect was observed for all tested insulin analogues.

## Discussion

Treatment of diabetic patients with insulin analogues has been shown to provide a more efficient, reproducible, and convenient therapy than regular insulin. The analogues may vary from insulin with respect to metabolic potency, stability or onset, and duration of action that is achieved by either sequence or secondary structural modifications. These changes may lead to an altered functional profile, emphasizing the importance of examining all steps in the action of an insulin analog in vitro and in vivo. Regarding the effects of insulin analogues in cardiomyocyte cell models no published data is available. Therefore, we compared the long–acting insulin analogues IGla and IDeg in cardiac cell models. Using this in vitro setting, we could show the absence of any difference in functional cardiac endpoint measurements and insulin signalling between the long-acting insulin analogues IGla and IDeg under steady state conditions.

Since Akt is a major element of the insulin signalling pathway, we first analysed the activation of Akt in ARVM and HL-1 cells after treatment with the different insulin analogues. Although our results show a slower onset of Akt activation in HL-1 cells treated with IDeg for 5 and 10 min compared to the other insulins (Fig. 1b, c), we did not observe a difference in Akt activation in HL-1 cells treated for 60 min and acutely treated ARVM (Fig. 1d and 2a). We speculate that a possible explanation for the slower onset of Akt phosphorylation in HL-1 cells might be the low binding affinity of IDeg towards the IR. In receptor binding studies using S-IR of both isoforms, the binding affinity for IDeg was found to be 13–15 % relative to human insulin [25]. The results from our indirect binding assays with the S-IR show a similar binding affinity of IDeg with ~18.6 % relative to human insulin. However, it should be noted that S-IR displays a less complex construct compared to M-IR. In these preparations the IR is surrounded by lipids and protein complexes. In M-IR preparations IDeg showed a binding affinity of ~4 % compared to Ins and ~13 % compared to IGlaM1. It could be possible that the fatty acid residue attached to IDeg interacts with other components of the membrane, and therefore leading to a reduced binding affinity towards the IR. The very low binding affinity of IDeg in M-IR (Fig. 1a) could be an explanation for the slower onset of action observed in HL-1 and Cor.4U^®^ cells. The results obtained in M-IR preparations with Ins and IGlaM1 and IDeg in S-IR are comparable to previously published data [21, 25].

Insulin-mediated Akt activation in the myocardium triggers a variety of processes, like glucose uptake, modification of calcium signalling and anti-apoptotic effects [26]. Even though under physiological conditions the main energy source for the heart is fatty acids, about one third is derived from glucose [27] and with increasing blood glucose and insulin level, glucose becomes the favoured substrate in the heart [2]. Therefore, we next analysed the ability of IGla and IDeg to stimulate glucose uptake under steady-state conditions in HL-1 cells, since we observed a full Akt activation with IDeg after 60 min. Under these conditions we observed no difference between IGla and IDeg in the insulin stimulated glucose uptake compared to Ins. We therefore conclude that IGla and IDeg are equipotent in regulating cardiac glucose consumption, at least under steady-state conditions.

Another important aspect of insulin function in cardiomyocytes is the positive inotropic effect which was already described in the 1920s by Visscher and Müller [28] and is due to an increased excitation–contraction coupling, which in turn is controlled by entry and release of Ca^2+^ from the sacroplasmatic reticulum (SR). Using insulin and the insulin analogues we observed a similar positive inotropic effect, as shown by comparable increased sarcomeric shortening, departure- and return-velocity in ARVM. Furthermore, we could show that the positive inotropic effect in ARVM is completely Akt dependent. The Akt dependency of cardiomyocyte contraction was shown previously by Graves et al. in HL-1 cells, where inhibition of Akt activation leads to decreased total [Ca^2+^]_i_, intracellular Ca^2+^ transients and membrane I_Ca_ [29]. Furthermore, Reinartz et al. [30] recently showed that Akt1 and Akt2 knockdown affected phosphorylation of proteins involved in regulation of heart contraction as well as relaxation and regulation of heart rate. Additionally, proteins involved in Ca^2+^ release and re-entry into the SR are affected (e.g. CaMKII or phospholamban, a direct target of Akt) [30, 31], which could be a possible explanation for the complete abrogation of the positive inotropic effect after Akt-inhibition. Insulin increases the beating–rate of cardiac muscle, but the underlying mechanism is controversially discussed in the literature. While some groups found evidence for insulin to directly increase the beating-rate in vivo [32, 33], others claimed that insulin acts on the nervous system and thereby leads to beta-adrenergic stimulation of the heart [34, 35]. In our in vitro experiments with Cor.4U^®^ cells we observed a slight but significant increase in beating-rate of Cor.4U^®^ cardiomyocytes for up to 6 h. As observed in HL–1 signalling, we measured a slower onset of action with IDeg. However under steady state conditions no differences between the different analogues could be detected. Together with the results from the ARVM contraction experiments we conclude that the increase in beating–rate of cardiomyocytes is at least partly independent of nervous system activity and directly affected by insulin itself.

Furthermore, insulin is known to reduce the damage of ischemia/reperfusion injury (IRI) in vivo as well as in vitro [36–39]. This damage is induced by massive production of reactive oxygen species (ROS) [40]. While diabetes per se is a risk factor for ischemic heart disease, the damage inflicted by IRI is even worse in diabetic patients [41]. Therefore, we aimed to mimic IRI in H9c2–E2 cardiomyocytes by challenging the cells with H_2_O_2_ with subsequent measurement of caspase 3/7 activity. To elucidate the potency of IGla and IDeg in prevention of caspase 3/7 activation during ROS treatment, we treated part of the cells with a combination of H_2_O_2_ and the respective insulin. With our results we were able to reproduce the protective effect of Ins during IRI and furthermore we were able to show that both, IGla and IDeg, have the same potency to prevent caspase 3/7 activation as Ins.

## Conclusion

In conclusion, the long-acting insulin analogues IGla and IDeg show no major differences in several cardiomyocyte in vitro models regarding insulin signalling, contractility parameters, beating–rate, glucose uptake, and protection from oxidative stress–induced caspase 3/7 activation under steady-state conditions. However, for IDeg we observed a slower onset of action in Akt phosphorylation in HL-1 cells as well as slower response to IDeg in human Cor.4U^®^ cardiomyocytes. Additionally, we observed very low binding affinities of IDeg in M-IR preparations. Whether these effects translate to the complex in vivo situation needs further evaluation.

## Additional files

10.1186/s12933-016-0410-9 Competition binding assay using solubilised IR. Solubilised IR preparations were used to analyse binding of Ins (blue), IGlaM1 (green) and IDeg (red) in a competition binding assay. Percentage of binding is normalised to maximum binding of [^125^I]-labelled human insulin. Data represent mean values ± SEM, n = 6–7.
10.1186/s12933-016-0410-9 Summary of competition binding assay using S-IR.
10.1186/s12933-016-0410-9 Phosphorylation of Akt(Thr^308^) in HL-1 cells. (A-C) HL-1 cells were used to assess the onset of insulin action by treatment with 200 nM for 5 (A); 10 (B) or 60 min (C) with insulin or insulin analogues. Phosphorylation of Akt(Thr^308^) was assessed by Western blot analysis. Data are normalised to tubulin levels. Representative blots are shown. Data represent mean values ± SEM, n = 3, *p < 0.05 vs. basal,^ #^p < 0.05 vs. IDeg. Regular insulin (Ins), insulin glargine (IGla), active metabolite of insulin glargine (IGlaM1), insulin degludec (IDeg).
10.1186/s12933-016-0410-9 Phosphorylation of Akt(Thr^308^) in adult rat ventricular myocytes. (A) Adult rat ventricular myocytes (ARVM) were used to assess the onset of insulin action by treatment with 100 nM for 10 min with insulin or insulin analogues. (B) ARVM were analysed either without pre-treatment (blank bars) or pre-treated with 10 µM of the specific Akt-inhibitor triciribine (filled bars) for 30 min. Subsequently, ARVM were treated with either 100 nM insulin or insulin analogues for 10 min to investigate the insulin signalling pathway after triciribine treatment. Phosphorylation of Akt(Thr^308^) was assessed by Western blot analysis. Data are normalised to GAPDH levels. Representative blots are shown. Data represent mean values ± SEM, n = 4–5, *p < 0.05 vs. basal. Regular insulin (Ins), insulin glargine (IGla), active metabolite of insulin glargine (IGlaM1), insulin degludec (IDeg).
10.1186/s12933-016-0410-9 Cell viability assay after triciribine treatment. Adult rat ventricular cardiomyocytes (ARVM) were stained with 0.1 % trypan blue in PBS for 5 min after treatment with 10 µM triciribine, subsequently random bright field images were taken and viable (white) and dead (blue) cells were quantified. (A–C) Representative bright field pictures of basal conditions (A), 10 µM triciribine treatment (B) and 200 µM H_2_O_2_ treatment (C). At least 400 cells per condition per experiment were counted. Scale bar = 200 µM. (D) Quantification of living and dead cells. Data represent mean values ± SEM, n = 3, *p < 0.05 vs. basal.

## Abbreviations

ARVM: adult rat ventricular myocytes
ANOVA: analysis of variance
CamKII: Ca^2+^/calmodulin-dependent protein kinase II
DMEM: Dulbecco’s modified eagle medium
ECL: enhanced chemiluminescence
FCS: fetal calf serum
FDA: American Food and Drug Administration
GAPDH: glyceraldehyde-3-phosphate dehydrogenase
H9c2-E2: H9c2 cells overexpressing the human insulin receptor
HRP: horse radish peroxidase
HSA: human serum albumin
IDeg: insulin degludec
IGla: insulin glargine
IGlaM1: active metabolite of insulin glargine
Ins: regular insulin
IR: insulin receptor
IRI: ischemia/reperfusion injury
M-IR: membrane embedded insulin receptor
PBS: phosphate buffered saline
PVDF: polyvinylidene fluoride
ROS: reactive oxygen species
S-IR: solubilised insulin receptor
SDS/PAGE: sodium dodecyl sulfate polyacrylamide gel electrophoresis
SEM: standard error of the mean
SPA: scintillation proximity assay
SR: sacroplasmatic reticulum
TBS: tris-buffered saline

## References

[1] Muniyappa R, Montagnani M, Koh KK, Quon MJ (2007). Cardiovascular actions of insulin. Endocr Rev.

[2] Bertrand L, Horman S, Beauloye C, Vanoverschelde JL (2008). Insulin signalling in the heart. Cardiovasc Res.

[3] Klein LJ, Visser FC (2010). The effect of insulin on the heart: part 1: effects on metabolism and function. Neth Heart J.

[4] ADA (2008). Standards of medical care in diabetes–2008. Diabetes Care.

[5] Rodbard HW, Gough S, Lane W, Korsholm L, Bretler DM, Handelsman Y (2014). Reduced risk of hypoglycemia with insulin degludec versus insulin glargine in patients with type 2 diabetes requiring high doses of Basal insulin: a meta-analysis of 5 randomized begin trials. Endocr Pract.

[6] Rosenstock J, Dailey G, Massi-Benedetti M, Fritsche A, Lin Z, Salzman A (2005). Reduced hypoglycemia risk with insulin glargine: a meta-analysis comparing insulin glargine with human NPH insulin in type 2 diabetes. Diabetes Care.

[7] Heinemann L, Linkeschova R, Rave K, Hompesch B, Sedlak M, Heise T (2000). Time-action profile of the long-acting insulin analog insulin glargine (HOE901) in comparison with those of NPH insulin and placebo. Diabetes Care.

[8] Rosskamp RH, Park G (1999). Long-acting insulin analogs. Diabetes Care.

[9] Kuerzel GU, Sandow J, Seipke G, Lang AM, Maas J, Skrzipczyk HJ. Kinetic and metabolite profile of insulin glargine (LANTUS^®^). Diabetologia. 2001;44.

[10] Kuerzel GU, Shukla U, Scholtz HE, Pretorius SG, Wessels DH, Venter C, Potgieter MA, Lang AM, Koose T, Bernhardt E (2003). Biotransformation of insulin glargine after subcutaneous injection in healthy subjects. Curr Med Res Opin.

[11] Jonassen I, Havelund S, Hoeg-Jensen T, Steensgaard DB, Wahlund PO, Ribel U (2012). Design of the novel protraction mechanism of insulin degludec, an ultra-long-acting basal insulin. Pharm Res.

[12] Hamilton-Wessler M, Ader M, Dea M, Moore D, Jorgensen PN, Markussen J, Bergman RN (1999). Mechanism of protracted metabolic effects of fatty acid acylated insulin, NN304, in dogs: retention of NN304 by albumin. Diabetologia.

[13] Kurtzhals P, Havelund S, Jonassen I, Kiehr B, Larsen UD, Ribel U, Markussen J (1995). Albumin binding of insulins acylated with fatty acids: characterization of the ligand-protein interaction and correlation between binding affinity and timing of the insulin effect in vivo. Biochem J.

[14] Myers SR, Yakubu-Madus FE, Johnson WT, Baker JE, Cusick TS, Williams VK, Tinsley FC, Kriauciunas A, Manetta J, Chen VJ (1997). Acylation of human insulin with palmitic acid extends the time action of human insulin in diabetic dogs. Diabetes.

[15] Gerstein HC, Bosch J, Dagenais GR, Diaz R, Jung H, Maggioni AP, Pogue J, Probstfield J, Ramachandran A, Riddle MC (2012). Basal insulin and cardiovascular and other outcomes in dysglycemia. N Engl J Med.

[16] Endocrinologic and metabolic drugs advisory committee meeting—insulin degludec and insulin degludec/aspart. http://www.fda.gov/downloads/advisorycommittees/committeesmeetingmaterials/drugs/endocrinologicandmetabolicdrugsadvisorycommittee/ucm330923.pdf.

[17] FDA approves two new drug treatments for diabetes mellitus. http://www.fda.gov/NewsEvents/Newsroom/PressAnnouncements/ucm464321.htm.

[18] Rask-Madsen C, Kahn CR (2012). Tissue-specific insulin signaling, metabolic syndrome, and cardiovascular disease. Arterioscler Thromb Vasc Biol.

[19] Claycomb WC, Lanson NA, Stallworth BS, Egeland DB, Delcarpio JB, Bahinski A, Izzo NJ (1998). HL-1 cells: a cardiac muscle cell line that contracts and retains phenotypic characteristics of the adult cardiomyocyte. Proc Natl Acad Sci USA.

[20] Uhlig M, Passlack W, Eckel J (2005). Functional role of Rab11 in GLUT4 trafficking in cardiomyocytes. Mol Cell Endocrinol.

[21] Sommerfeld MR, Muller G, Tschank G, Seipke G, Habermann P, Kurrle R, Tennagels N (2010). In vitro metabolic and mitogenic signaling of insulin glargine and its metabolites. PLoS One.

[22] Hansen BF, Glendorf T, Hegelund AC, Lundby A, Lutzen A, Slaaby R, Stidsen CE (2012). Molecular characterisation of long-acting insulin analogues in comparison with human insulin, IGF-1 and insulin X10. PLoS One.

[23] Eckel J, Pandalis G, Reinauer H (1983). Insulin action on the glucose transport system in isolated cardiocytes from adult rat. Bio Chem J.

[24] Scott CW, Zhang X, Abi-Gerges N, Lamore SD, Abassi YA, Peters MF (2014). An impedance-based cellular assay using human iPSC-derived cardiomyocytes to quantify modulators of cardiac contractility. Toxicol Sci.

[25] Nishimura E, Sørensen AR, Hansen BF, Stidsen CE, Olsen GS, Schaäffer L (2010). Insulin degludec: a new ultra-long, basal insulin designed to maintain full metabolic effect while minimizing mitogenic potential. Diabetologia.

[26] Sussman MA, Volkers M, Fischer K, Bailey B, Cottage CT, Din S, Gude N, Avitabile D, Alvarez R, Sundararaman B (2011). Myocardial AKT: the omnipresent nexus. Physiol Rev.

[27] Lopaschuk GD, Ussher JR, Folmes CD, Jaswal JS, Stanley WC (2010). Myocardial fatty acid metabolism in health and disease. Physiol Rev.

[28] Visscher MB, Muller EA (1927). The influence of insulin upon the mammalian heart. J Physiol.

[29] Graves BM, Simerly T, Li C, Williams DL, Wondergem R (2012). Phosphoinositide-3-kinase/akt - dependent signaling is required for maintenance of [Ca(2 +)](i), I(Ca), and Ca(2 +) transients in HL-1 cardiomyocytes. J Biomed Sci.

[30] Reinartz M, Raupach A, Kaisers W, Godecke A (2014). AKT1 and AKT2 induce distinct phosphorylation patterns in HL-1 cardiac myocytes. J Proteome Res.

[31] Catalucci D, Latronico MV, Ceci M, Rusconi F, Young HS, Gallo P, Santonastasi M, Bellacosa A, Brown JH, Condorelli G (2009). Akt increases sarcoplasmic reticulum Ca2 + cycling by direct phosphorylation of phospholamban at Thr17. J Biol Chem.

[32] Jacobsen F, Christensen NJ (1979). Stimulation of heart rate by insulin: uninfluenced by beta-adrenergic receptor blockade in rabbits. Scand J Clin Lab Invest.

[33] Keen HL, Brands MW, Alonso-Galicia M, Hall JE (1996). Chronic adrenergic receptor blockade does not prevent hyperinsulinemia-induced hypertension in rats. Am J Hypertens.

[34] Anderson EA, Hoffman RP, Balon TW, Sinkey CA, Mark AL (1991). Hyperinsulinemia produces both sympathetic neural activation and vasodilation in normal humans. J Clin Invest.

[35] Siani A, Strazzullo P, Giorgione N, De LA, Mancini M (1990). Insulin-induced increase in heart rate and its prevention by propranolol. Eur J Clin Pharmacol.

[36] Ji L, Fu F, Zhang L, Liu W, Cai X, Zhang L, Zheng Q, Zhang H, Gao F (2010). Insulin attenuates myocardial ischemia/reperfusion injury via reducing oxidative/nitrative stress. Am J Physiol Endocrinol Metab.

[37] Shi YF, Liu N, Li YX, Song CL, Song XJ, Zhao Z, Liu B (2015). Insulin protects H9c2 rat cardiomyoblast cells against hydrogen peroxide-induced injury through upregulation of microRNA-210. Free Radic Res.

[38] Wong VW, Mardini M, Cheung NW, Mihailidou AS (2011). High-dose insulin in experimental myocardial infarction in rabbits: protection against effects of hyperglycaemia. J Diabetes Complicat.

[39] Xing W, Yan W, Fu F, Jin Y, Ji L, Liu W, Wang L, Lv A, Duan Y, Zhang J (2009). Insulin inhibits myocardial ischemia-induced apoptosis and alleviates chronic adverse changes in post-ischemic cardiac structure and function. Apoptosis.

[40] Braunersreuther V, Jaquet V (2012). Reactive oxygen species in myocardial reperfusion injury: from physiopathology to therapeutic approaches. Curr Pharm Biotechnol.

[41] Ferdinandy P, Schulz R, Baxter GF (2007). Interaction of cardiovascular risk factors with myocardial ischemia/reperfusion injury, preconditioning, and postconditioning. Pharmacol Rev.

