# Supplementary material for: Effect of the long-acting insulin analogues glargine and degludec on cardiomyocyte cell signalling and function
Source: Cardiovasc Diabetol. 2016 Jul 15;15:96. doi: 10.1186/s12933-016-0410-9 (PMC4946153; doi:10.1186/s12933-016-0410-9)
Supplement: Supplementary file 1 — 10.1186/s12933-016-0410-9 Competition binding assay using solubilised IR. Solubilised IR preparations were used to analyse binding of Ins (blue), IGlaM1 (green) and IDeg (red) in a competition binding assay. Percentage of binding is normalised to maximum binding of [125I]-labelled human insulin. Data represent mean values ± SEM, n = 6–7. [file 12933_2016_410_MOESM1_ESM.docx]

**
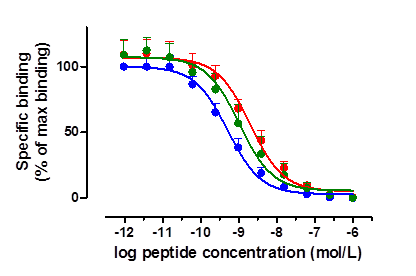
**

**Supplementary Figure 1: Competition binding assay using solubilised insulin receptor.** Solubilised insulin receptor preparations were used to analyse binding of Ins (blue), IGlaM1 (green) and IDeg (red) in a competition binding assay. Percentage of binding is normalised to maximum binding of [^125^I]-labelled human insulin. Data represent mean values ± SEM, n = 6-7.
