# Supplementary material for: Effect of the long-acting insulin analogues glargine and degludec on cardiomyocyte cell signalling and function
Source: Cardiovasc Diabetol. 2016 Jul 15;15:96. doi: 10.1186/s12933-016-0410-9 (PMC4946153; doi:10.1186/s12933-016-0410-9)
Supplement: Supplementary file 2 — 10.1186/s12933-016-0410-9 Summary of competition binding assay using S-IR. [file 12933_2016_410_MOESM2_ESM.docx]

**Supplementary Table 1: Summary of competition binding assay using S-IR**

| Insulin | IR affinity  IC_50_ (nmol/L) | p-value vs. Ins |
| --- | --- | --- |
| Ins | 0.58 ± 0.10 | - |
| IGlaM1 | 2.09 ± 0.74 | 0.10 |
| IDeg | 3.12 ± 0.42 | <0.01 |

Data represent means ± SEM. All insulins were used at least six times on different days. IC_50_ were obtained in quadruplicates per insulin and averaged for each experiment. Regular insulin (Ins), active metabolite of glargine (IGlaM1), insulin degludec (IDeg)
