# Supplementary material for: Effect of the long-acting insulin analogues glargine and degludec on cardiomyocyte cell signalling and function
Source: Cardiovasc Diabetol. 2016 Jul 15;15:96. doi: 10.1186/s12933-016-0410-9 (PMC4946153; doi:10.1186/s12933-016-0410-9)
Supplement: Supplementary file 3 — 10.1186/s12933-016-0410-9 Phosphorylation of Akt(Thr308) in HL-1 cells. (A-C) HL-1 cells were used to assess the onset of insulin action by treatment with 200 nM for 5 (A); 10 (B) or 60 min (C) with insulin or insulin analogues. Phosphorylation of Akt(Thr308) was assessed by Western blot analysis. Data are normalised to tubulin levels. Representative blots are shown. Data represent mean values ± SEM, n = 3, *p < 0.05 vs. basal, #p < 0.05 vs. IDeg. Regular insulin (Ins), insulin glargine (IGla), active metabolite of insulin glargine (IGlaM1), insulin degludec (IDeg). [file 12933_2016_410_MOESM3_ESM.docx]

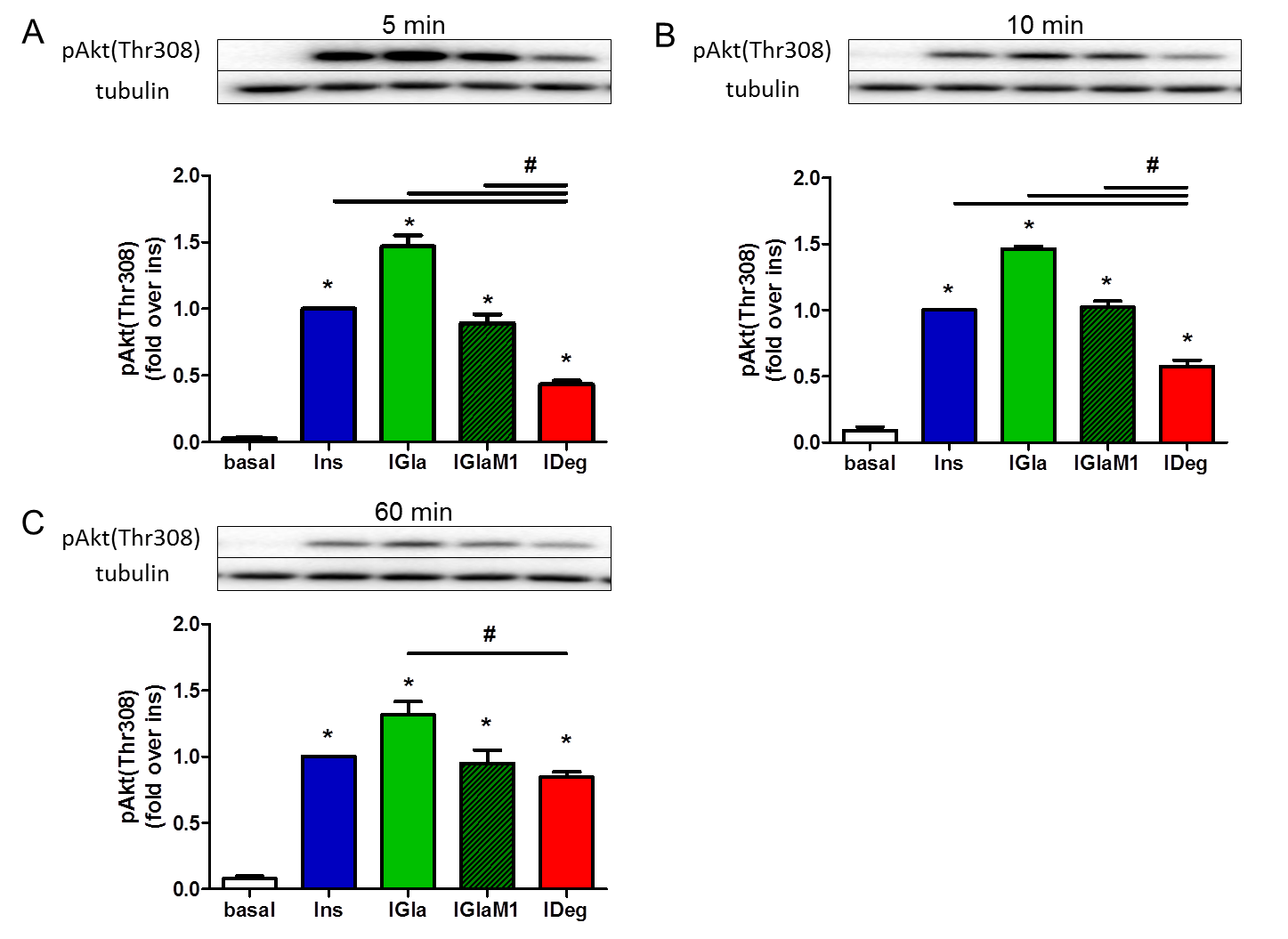


**Supplementary Figure 2**: **Phosphorylation of Akt(Thr^308^) in HL-1 cells**. (A-C) HL-1 cells were used to assess the onset of insulin action by treatment with 200 nM for 5 (A); 10 (B) or 60 min (C) with insulin or insulin analogues. Phosphorylation of Akt(Thr^308^) was assessed by Western blot analysis. Data are normalised to tubulin levels. Representative blots are shown. Data represent mean values ± SEM, n = 3, *p<0.05 vs. basal, #p<0.05 vs. IDeg. Regular insulin (Ins), insulin glargine (IGla), active metabolite of insulin glargine (IGlaM1), insulin degludec (IDeg).
