# Supplementary material for: Effect of the long-acting insulin analogues glargine and degludec on cardiomyocyte cell signalling and function
Source: Cardiovasc Diabetol. 2016 Jul 15;15:96. doi: 10.1186/s12933-016-0410-9 (PMC4946153; doi:10.1186/s12933-016-0410-9)
Supplement: Supplementary file 4 — 10.1186/s12933-016-0410-9 Phosphorylation of Akt(Thr308) in adult rat ventricular myocytes. (A) Adult rat ventricular myocytes (ARVM) were used to assess the onset of insulin action by treatment with 100 nM for 10 min with insulin or insulin analogues. (B) ARVM were analysed either without pre-treatment (blank bars) or pre-treated with 10 µM of the specific Akt-inhibitor triciribine (filled bars) for 30 min. Subsequently, ARVM were treated with either 100 nM insulin or insulin analogues for 10 min to investigate the insulin signalling pathway after triciribine treatment. Phosphorylation of Akt(Thr308) was assessed by Western blot analysis. Data are normalised to GAPDH levels. Representative blots are shown. Data represent mean values ± SEM, n = 4–5, *p < 0.05 vs. basal. Regular insulin (Ins), insulin glargine (IGla), active metabolite of insulin glargine (IGlaM1), insulin degludec (IDeg). [file 12933_2016_410_MOESM4_ESM.docx]

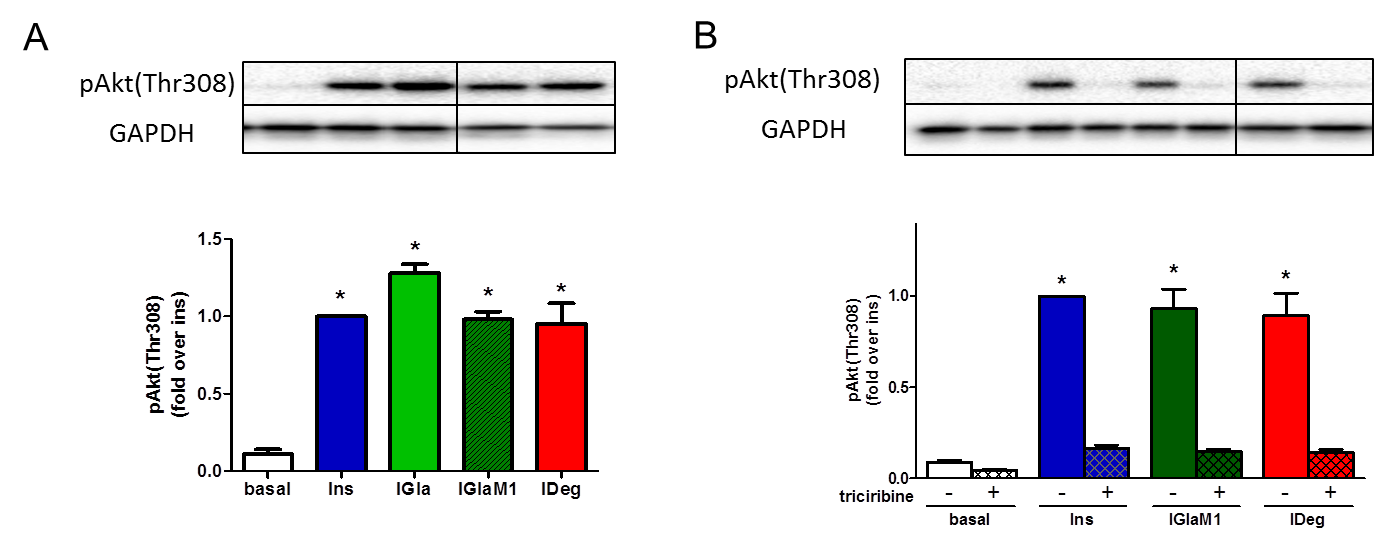


**Supplementary Figure 3: Phosphorylation of Akt(Thr^308^) in adult rat ventricular myocytes.** (A) Adult rat ventricular myocytes (ARVM) were used to assess the onset of insulin action by treatment with 100 nM for 10 min with insulin or insulin analogues. (B) ARVM were analysed either without pre-treatment (blank bars) or pre-treated with 10 µM of the specific Akt-inhibitor triciribine (filled bars) for 30 min. Subsequently, ARVM were treated with either 100 nM insulin or insulin analogues for 10 minutes to investigate the insulin signalling pathway after triciribine treatment. Phosphorylation of Akt(Thr^308^) was assessed by Western blot analysis. Data are normalised to GAPDH levels. Representative blots are shown. Data represent mean values ± SEM, n = 4‑5, *p<0.05 vs. basal. Regular insulin (Ins), insulin glargine (IGla), active metabolite of insulin glargine (IGlaM1), insulin degludec (IDeg)
