# Supplementary material for: Effect of the long-acting insulin analogues glargine and degludec on cardiomyocyte cell signalling and function
Source: Cardiovasc Diabetol. 2016 Jul 15;15:96. doi: 10.1186/s12933-016-0410-9 (PMC4946153; doi:10.1186/s12933-016-0410-9)
Supplement: Supplementary file 5 — 10.1186/s12933-016-0410-9 Cell viability assay after triciribine treatment. Adult rat ventricular cardiomyocytes (ARVM) were stained with 0.1 % trypan blue in PBS for 5 min after treatment with 10 µM triciribine, subsequently random bright field images were taken and viable (white) and dead (blue) cells were quantified. (A–C) Representative bright field pictures of basal conditions (A), 10 µM triciribine treatment (B) and 200 µM H2O2 treatment (C). At least 400 cells per condition per experiment were counted. Scale bar = 200 µM. (D) Quantification of living and dead cells. Data represent mean values ± SEM, n = 3, *p < 0.05 vs. basal. [file 12933_2016_410_MOESM5_ESM.docx]

**
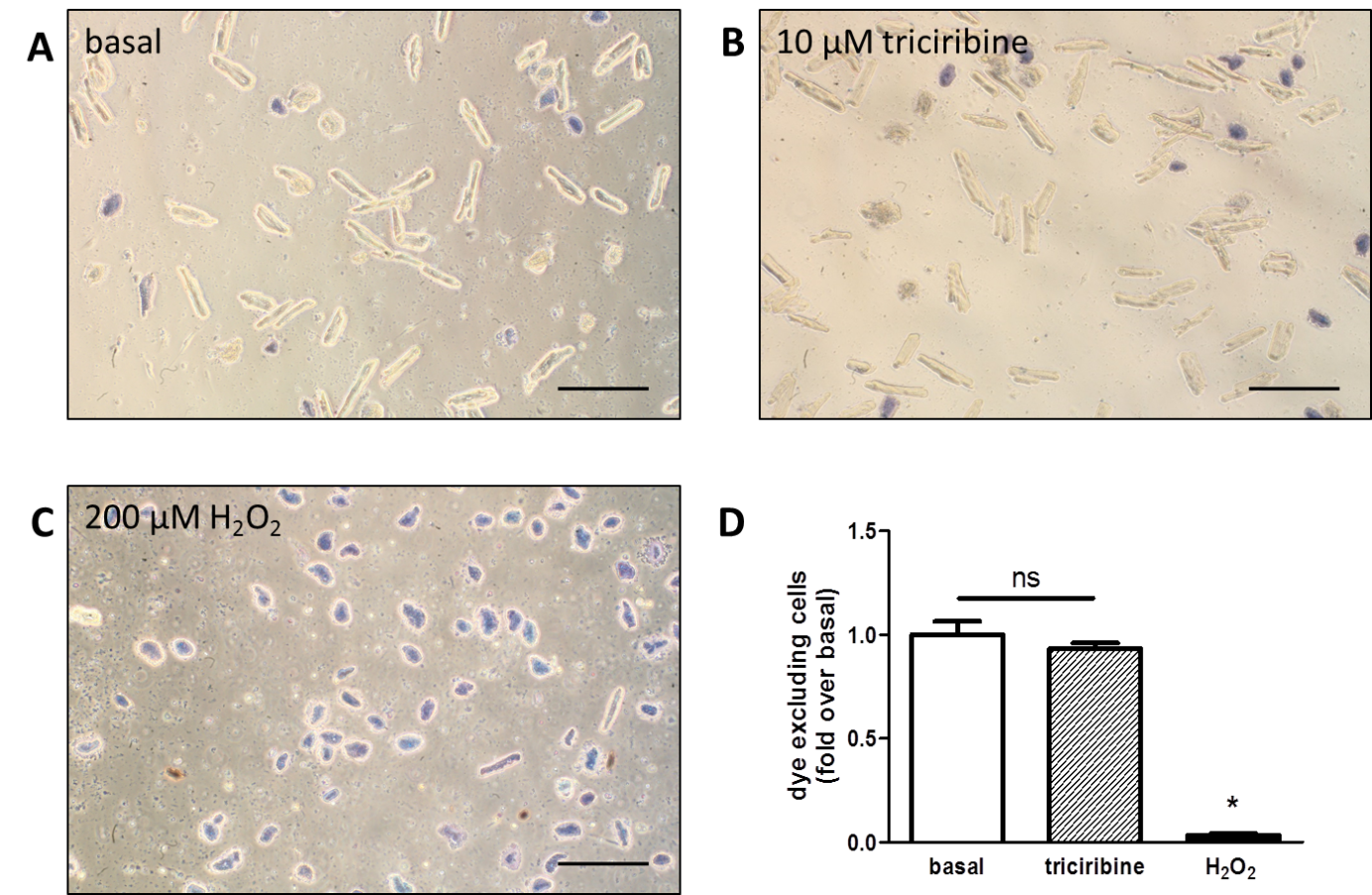
**

**Supplementary Figure 4: Cell viability assay after triciribine treatment.** Adult rat ventricular cardiomyocytes (ARVM) were stained with 0.1 % trypan blue in PBS for 5 minutes after treatment with 10 µM triciribine, subsequently random bright field images were taken and viable (white) and dead (blue) cells were quantified. (A‑C) Representative bright field pictures of basal conditions (A), 10 µM triciribine treatment (B) and 200 µM H_2_O_2_ treatment (C). At least 400 cells per condition per experiment were counted. Scale bar = 200 µM. (D) Quantification of living and dead cells. Data represent mean values ± SEM, n = 3, *p<0.05 vs. basal.
